# Marine bacteria from the French Atlantic coast displaying high forming-biofilm abilities and different biofilm 3D architectures

**DOI:** 10.1186/s12866-015-0568-4

**Published:** 2015-10-24

**Authors:** Ibtissem Doghri, Sophie Rodrigues, Alexis Bazire, Alain Dufour, David Akbar, Valérie Sopena, Sophie Sablé, Isabelle Lanneluc

**Affiliations:** Université de La Rochelle, UMR 7266 CNRS-LIENSs, Bât. Marie Curie, Av. Michel Crépeau, 17042, La Rochelle cedex 01, France; Université de Bretagne-Sud, EA 3884, LBCM, IUEM, 56100, Lorient, France

**Keywords:** Marine bacteria, Biofilm, Abiotic surfaces, Biofilm 3D structures

## Abstract

**Background:**

Few studies have reported the species composition of bacterial communities in marine biofilms formed on natural or on man-made existing structures. In particular, the roles and surface specificities of primary colonizers are largely unknown for most surface types. The aim of this study was to obtain potentially pioneering bacterial strains with high forming-biofilm abilities from two kinds of marine biofilms, collected from two different surfaces of the French Atlantic coast: an intertidal mudflat which plays a central role in aquaculture and a carbon steel structure of a harbour, where biofilms may cause important damages.

**Results:**

A collection of 156 marine heterotrophic aerobic bacteria isolated from both biofilms was screened for their ability to form biofilms on polystyrene 96-well microtiter plates. Out of 25 strains able to build a biofilm in these conditions, only four bacteria also formed a thick and stable biofilm on glass surfaces under dynamic conditions. These strains developed biofilms with four different three - dimensional architectures when observed by confocal laser scanning microscopy: *Flavobacterium* sp. II2003 biofilms harboured mushroom-like structures, *Roseobacter* sp. IV3009 biofilms were quite homogeneous, *Shewanella* sp. IV3014 displayed hairy biofilms with horizontal fibres, whereas *Roseovarius* sp. VA014 developed heterogeneous and tousled biofilms.

**Conclusions:**

This work led for the first time to the obtaining of four marine bacterial strains, potentially pioneering bacteria in marine biofilms, able to adhere to at least two different surfaces (polystyrene and glass) and to build specific 3D biofilms. The four selected strains are appropriate models for a better understanding of the colonization of a surface as well as the interactions that can occur between bacteria in a marine biofilm, which are crucial events for the initiation of biofouling.

## Background

Biofilms are generally considered as surface-associated microorganism communities encased in a hydrated polymeric matrix [1]. In marine environment, most of the solid man-made structures as well as natural surfaces are covered by microbial biofilms. Together with diatoms, bacteria constitute the major components of biofilms occurring in the marine environment [2]. Furthermore, all types of bacteria can form biofilms, making this sedentary lifestyle their favourite mode of existence in nature [3]. This mechanism is described as considerably important for survival of marine bacteria by providing a favourable environment [4]. This lifestyle, compared to the planktonic one, indeed improves access to nutrients and protects against stress, antibiotics and predators [1, 5].

Quickly after immersion of a clean surface in the sea, microorganisms colonize it and subsequently develop biofilms with highly diverse three-dimensional (3D) structures which can include channels allowing the flow of liquids, nutrients and wastes [6–8]. The early stages of biofilm formation are based on the interactions of free-living bacteria with the surface which generates an initial layer of microorganisms and polymers [5]. Thereafter, growth of the primary colonizing bacteria changes the surface characteristics of the substratum, rendering it suitable for subsequent colonization by other microorganisms. Finally, the mature biofilm community is formed through synergistic and/or competitive interactions [5].

Few studies have reported the species composition of bacterial communities in marine biofilms. In particular, the roles and surface specificities of primary colonizers are largely unknown for most surface types [9]. The early-stage biofilms were dominated by the same major classes of bacteria that were most abundant in planktonic communities, with the latter demonstrating a higher diversity when compared with that of biofilm bacteria [9, 10]. The Alphaproteobacteria and Gammaproteobacteria were recognized as the pioneering microorganisms in marine biofilm formation [2, 5, 9–11]. Then, Acidobacteria, Actinobacteria, Bacteroidetes, Chloroflexi, Cyanobacteria, Firmicutes, Planctomycetes, Verrucomicrobia and Beta, Delta and Epsilon groups of Proteobacteria were identified as minor phyla also belonging to these biofilms [5, 8–10, 12–14]. However, the bacterial composition of early-stage biofilms may be affected by the physicochemical properties of the solid surface [5, 10] and by the variation of environmental conditions due for example to the seasons or the characteristics of the immersion sites [2].

In the present work, we studied bacteria of two original types of marine biofilms from the French Atlantic coast, displaying different characteristics. The first one is a non-permanent benthic biofilm sampled from an intertidal mudflat, which plays a central role in the production of oysters. Indeed, oyster larvae can directly digest and assimilate bacterial carbon [15]. Moreover, this biofilm indirectly feeds the planktonic trophic network through re-suspension of the biofilm in the water column. However, current knowledge on the structure as well as the functioning of this biofilm is largely conceptual and theoretical. The second one is a permanent biofilm formed on carbon steel structures immersed in a French Atlantic harbour and involved in the early stages of the biocorrosion phenomenon [16]. In seawater, complex biofilms closely linked with corrosion products quickly develop on metallic structures. This can influence the deterioration of metal by microorganism activity, thus causing great damages to harbour infrastructures, resulting in economic losses. To date, the initiation of the microbiologically-influenced corrosion processes remains unclear.

Understanding the initial stage of marine biofilm formation is highly important to explain the biofilm formation phenomenon. Unlike the studies in which the structure of pioneering communities, developed on immersed artificial surfaces in seawater, is directly investigated [9, 14], our approach was first to build a collection of culturable marine bacteria isolated from two kinds of biofilms and then to screen for the ability of each isolate to adhere to artificial surfaces and to form biofilms under the same controlled conditions. The objective of this work was to obtain model strains with high forming-biofilm abilities, suitable for further experiments which would allow a better understanding of the colonization of a surface as well as the interactions that can occur between bacteria in a marine biofilm.

## Results

### Screening of bacteria for their ability to adhere and to form biofilms on polystyrene surfaces

**Fig. 1:**
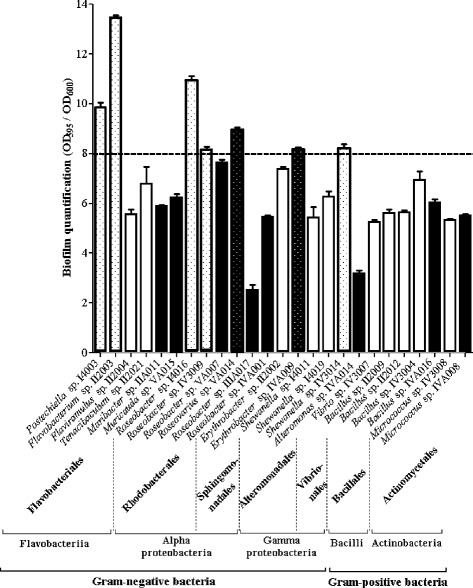
Quantification of bacterial biofilm formation on polystyrene microtiter plates under static conditions. Bacteria were isolated from intertidal mudflat biofilms (white bars) and from corrosion product-microorganism composite biofilms developed on harbor metallic structures (black bars). After 24 h of growth, single-species biofilms were quantified with crystal violet and the ratios of OD_595_ (cells grown in biofilm)/OD_600_ (planktonic cells) were calculated. The ratios are represented on the *y* axis. Dotted bars: bacteria with a ratio OD_595_/OD_600_ >8. Bars represent means ± standard deviations for three replicates

In order to efficiently select strains able to adhere and to form biofilms, the whole bacterial collection of 156 heterotrophic aerobic bacteria, isolated as described in the “Methods” section, was first screened with a rapid method, based on crystal violet staining of biofilms formed in 96-well microtiter plates (polystyrene surfaces). The bacteria that efficiently formed biofilms onto this surface after a 2 h adhesion step and 24 h of growth were identified by 16S rDNA sequencing. This screening revealed that the biofilm formation ability was very variable according to the bacterial strains (Fig. 1).

**Fig. 2:**
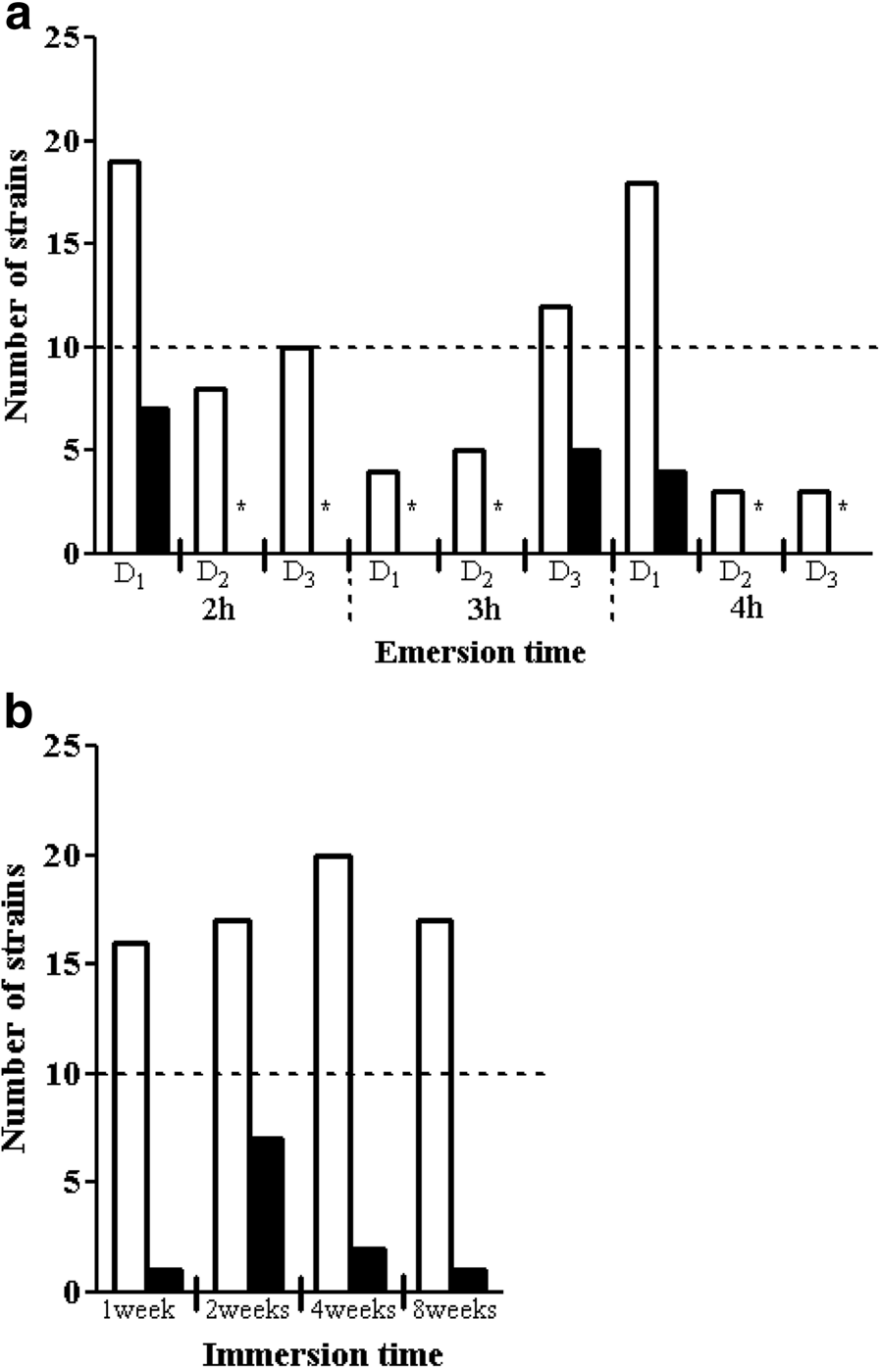
Proportion of bacterial strains able to form biofilms on polystyrene microtiter plates under static conditions. Results are presented according to the emersion time for bacteria isolated from mudflat biofilms (**a**) or the immersion time for bacteria isolated from corrosion product-microorganism composite biofilms (**b**). Mudflat sampling was performed three times at low tide during three days (D_1_, D_2_, and D_3_). White bars: number of strains tested. Black bars: number of forming-biofilm strains. *: no forming-biofilm strain detected

Under our experimental conditions, out of 86 isolates from the intertidal mudflat biofilms, 15 strains were able to form a biofilm with a ratio of cel1s grown in biofilm/planktonic cells higher than 2 (Fig. 1). These biofilm-forming bacteria were distributed in 5 bacterial classes: Flavobacteriia (27 %), Gammaproteobacteria (27 %), Alphaproteobacteria (20 %), Bacilli (20 %) and Actinobacteria (6 %). The *Flavobacterium* sp. II2003 strain, with a ratio of 13, showed the best ability to form a biofilm on polystyrene. Other bacteria displayed a strong ability to form a biofilm on polystyrene: *Postechiella* sp. I4003, *Roseobacter* sp. I4016 and IV3009 and *Shewanella* sp. IV3014 showed a ratio higher than 8 (Fig. 1). The proportion of benthic bacterial strains able to form biofilms according to the sampling time at low tide is presented in Fig. 2a. Bacteria forming a biofilm were found at all emersion times (from 2 to 4 h). However, when less than 10 strains were isolated from a sample, no biofilm-forming bacterium was detected in this sample, whatever the emersion time (Fig. 2a).

Concerning the bacteria isolated from biofilms developed on corroded carbon steel immersed in sea water, 10 strains among the 70 isolates were able to form biofilms after 24 h (with a ratio of cells grown in biofilm/planktonic cells higher than 2) (Fig. 1). These strains were affiliated to the same taxonomic groups as the benthic bacteria, but the proportion of bacteria from each class varied: 50 % Alphaproteobacteria, 20 % Flavobacteriia, 10 % Gammaproteobacteria, 10 % Bacilli and 10 % Actinobacteria. Under our experimental conditions, the ratios of cel1s grown in biofilm/planktonic cells obtained for these bacteria were lower than for the benthic bacteria. The ratio of 9, for the *Roseovarius* sp. VA014 strain, was the highest value obtained for bacteria isolated from corroded structures (Fig. 1). *Erythrobacter* sp. IVA009 was also interesting with a ratio higher than 8 (Fig. 1). The results presented in Fig. 2b show that bacteria able to form a biofilm on polystyrene were found in all samples, but the highest number was isolated from the steel immersed for 2 weeks.

In conclusion, this first screening allowed us to detect 15 benthic bacteria and 10 bacteria from corroded structures able to develop a biofilm in 96-well polystyrene microplates.

### Ability of the selected strains to adhere and to form biofilms under static conditions on glass surfaces

**Fig. 3:**
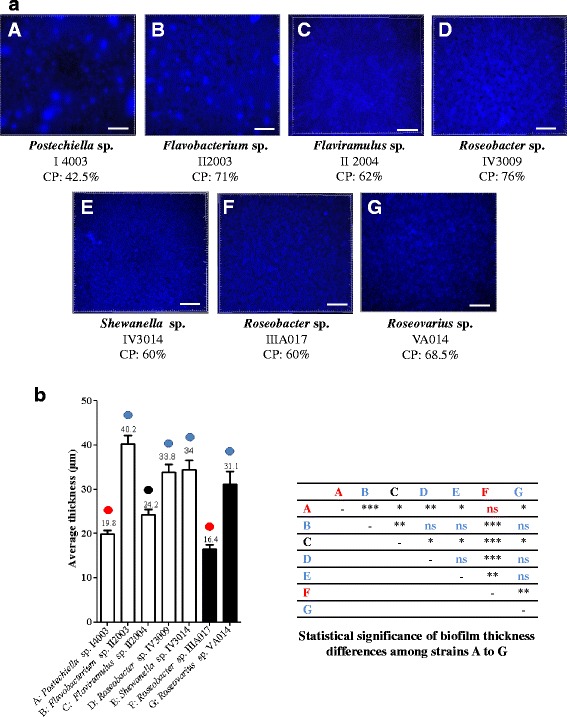
Fluorescence microscopic 3D reconstitutions and quantification of biofilms formed on glass surfaces under static conditions. After 24 h of growth under static conditions, biofilms were stained with DAPI. Microscopic 3D images were reconstituted (**a**), the average thicknesses of the biofilms were determined and the differences between them were statistically tested (**b**). **A**, **B**, **C**, **D**, **E**: bacterial isolates from mudflat biofilms. **F**, **G**: bacterial isolates from corrosion product-microorganism composite biofilms. Scale bar: 200 μm. CP: percentage of colonized surface. These values are averages of data from three independent experiments, with standard deviations lower than 10 % of each value. *: *p* < 0.05; **: *p* < 0.01; ***: *p* < 0.001. ns: not significant. Circles with the same color indicate bacteria with no significant biofilm thickness differences. Blue circles: thickest biofilms. Red circles: thinnest biofilms. Black circle: intermediate thickness

The above screening method in polystyrene microplates was rapid and convenient to detect the bacterial ability to form biofilms, but did not provide any structural information on these biofilms. To get this kind of information and thus study more accurately stable biofilms, the experiments had to be performed in dynamic conditions with biofilm observation by confocal laser scanning microscopy. Such observations required glass surfaces, and it was uncertain whether strains able to develop a biofilm on polystyrene would also be able to do it on glass. Since the biofilm study in dynamic conditions was labour intensive and time consuming, all bacteria that formed biofilms on polystyrene were then screened for their ability to form a biofilm on glass surfaces, first of all in static conditions. After a 2 h adhesion step and 24 h of growth, the biofilm was stained with DAPI, and microscopic fluorescence observations were performed to detect the strains behaviour on the glass surface. Among the previously selected bacteria (15 benthic bacteria and 10 bacteria isolated from corroded structures), only the *Postechiella* sp. I4003, *Flavobacterium* sp. II2003, *Flaviramulus* sp. II2004, *Roseobacter* sp. IV3009 and *Shewanella* sp. IV3014 benthic bacteria and the *Roseobacter* sp. IIIA017 and *Roseovarius* sp. VA014 strains from corroded structures were able to form biofilms under these conditions (Fig. 3). Microscopic observations of their biofilms showed a high percentage of colonized surfaces, from 42.5 % for *Postechiella* sp. I4003 to 76 % for *Roseobacter* sp. IV3009 (Fig. 3a). On the bases of the biofilm structures, two types of biofilms could be distinguished (Fig. 3a). The *Postechiella* sp. I4003, *Flavobacterium* sp. II2003 and *Roseobacter* sp. IV3009 biofilms were very heterogeneous and contained large cell aggregates whereas the *Roseobacter* IIIA017, *Roseovarius* sp. VA014, *Flaviramulus* sp. II2004 and *Shewanella* sp. IV3014 biofilms contained more evenly distributed cells. To classify the strains, bacteria were gathered when they exhibited no significant biofilm thickness differences. Thus, *Flavobacterium* sp. II2003, *Roseobacter* sp. IV3009, *Shewanella* sp. IV3014 and *Roseovarius* sp. VA014 were grouped. They built significantly thicker biofilms (40.2 μm, 33.8 μm, 34 μm and 31.1 μm respectively, Fig. 3b). *Roseobacter* sp. IIIA017 and *Flavobacterium* sp. I4003 biofilms were significantly thinner than all other biofilms, with an average of 16.4 μm and 19.8 μm respectively (Fig. 3b), and formed another group. Finally, *Flaviramulus* sp. II2004 biofilm exhibited an intermediate average thickness (24.2 μm), significantly different from all other strain biofilms (Fig. 3b).

### Study of the bacterial biofilm structures under dynamic conditions in flow cells

Through the previous steps, seven bacteria have been selected for their capability to form a thick biofilm under static conditions on polystyrene as well as glass surfaces. These bacteria were then studied under dynamic conditions, to further investigate strains able to develop stable biofilms. Thus, bacterial biofilms were grown on glass slides in three-channel flow cells and observed by confocal laser scanning microscopy after staining with the Syto 61 fluorescent dye.

**Fig. 4:**
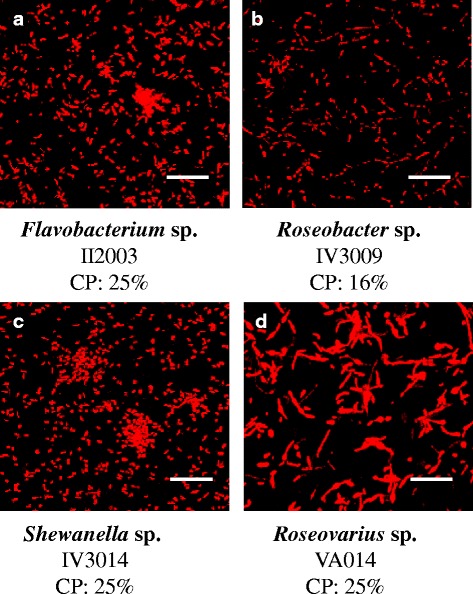
Confocal laser scanning microscopy images of attached cells after 2 h of adhesion on glass surfaces. Bacteria were allowed to attach into the flow cells during 2 h in artificial seawater without flow. Syto 61 red was used to stain the attached cells. **a**, **b**, **c**: bacterial isolates from mudflat biofilms. **d**: bacterial isolate from corrosion product-microorganism composite biofilms. Scale bar: 47μm. CP: percentage of colonized surface. These values are averages of data from three independent experiments, with standard deviations lower than 10 % of each value

**Fig. 5:**
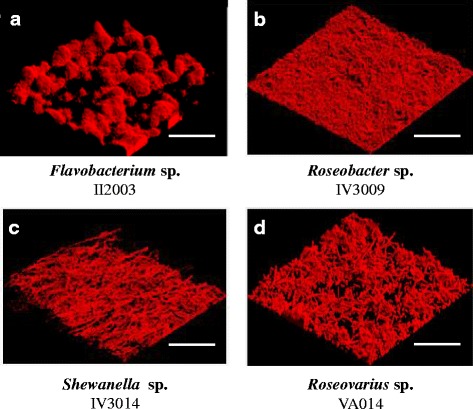
Confocal laser scanning microscopy images of single-species biofilms formed after 24 h of growth on glass surfaces under dynamic conditions. Biofilms were grown on glass surfaces in flow cells, at 22° C for 24 h, under a flow of Zobell medium. Bacteria were stained with Syto 61 red. **a**, **b**, **c**: 3D views of biofilms of bacterial isolates from mudflat biofilms. **d**: 3D view of a biofilm of the bacterial isolate from corrosion product-microorganism composite biofilms. Each image is representative of 10 observations. Scale bar: 67.3μm

The seven strains were able to attach onto the glass slide during a 2 h adhesion step in artificial seawater without flow, but the biofilms of three strains were not sufficiently stable and only four strains (*Flavobacterium* sp. II2003, *Roseobacter* sp. IV3009, *Shewanella* sp*.* IV3014 and *Roseovarius* sp. VA014) were able to form biofilms after 24 h of growth under a continuous culture medium flow. The microscopic observations of these strains are shown in Figs. 4 and 5. After the adhesion step, *Flavobacterium* sp. II2003, *Shewanella* sp. IV3014 and *Roseovarius* sp. VA014 began to form aggregates or microcolonies, whereas *Roseobacter* sp. IV3009 cells were more individually attached (Fig. 4). *Roseovarius* sp.VA014 cells were more filamentous. About 25 % of the glass surfaces were covered for all strains, except for the *Roseobacter* sp. IV3009 strain, which exhibited a significantly (*p* < 0.05) lower percentage of colonized surface (16 %, Fig. 4). After 24 h of biofilm growth, four different 3D architectures were observed (Fig. 5). *Flavobacterium* sp*.* II2003 biofilms harbouring numerous mushroom-like structures with a non-uniform distribution are reminiscent of biofilms of the well-known *Pseudomonas aeruginosa* model [17, 18].

**Fig. 6:**
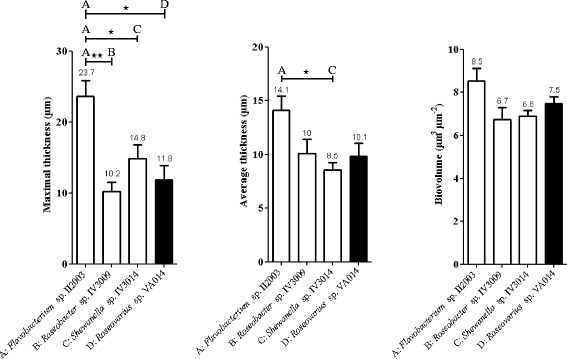
COMSTAT analyses of biofilms formed on glass surfaces, after 24 h of growth under dynamic conditions. **A**, **B**, **C**: bacterial isolates from mudflat biofilms (white bars). **D**: bacterial isolate from corrosion product-microorganism composite biofilms (black bars). Significant differences were only observed in isolate pairs **A-B**, **A-B**, **A–D** for maximal thickness, **A**–**C** for average thickness and are indicated by * (*p* < 0.05) or ** (*p* < 0.01) on the upper part of the Figure. In all the other cases, the differences were not significant

The *Flavobacterium* sp*.* II2003 biofilms presented significantly higher maximal thicknesses compared to biofilms of the three other strains, due to the mushroom-like structures, but their average thicknesses were only significantly higher than those of the *Shewanella* sp*.* IV3014 biofilms (Fig. 6). *Shewanella* sp*.* IV3014 displayed hairy biofilms with horizontal fibres, whereas *Roseovarius* sp. VA014 developed heterogeneous and tousled biofilms with cell aggregates (Fig. 5). *Roseobacter* sp. IV3009 biofilms were quite homogeneous with a bacterial distribution covering the entire surface (Fig. 5). The average and maximal thicknesses of *Roseobacter* sp. IV3009 biofilms were the same (10 μm, Fig. 6), confirming the regular distribution of cells. No significant differences were observed between *Shewanella* sp*.* IV3014, *Roseovarius* sp. VA014 and *Roseobacter* sp. IV3009 for the average and the maximal biofilm thicknesses (Fig. 6). Similarly, the biovolumes of all 4 biofilms were not significantly different from each other.

In conclusion, only four strains, *Flavobacterium* sp. II2003, *Roseobacter* sp. IV3009, *Shewanella* sp*.* IV3014 from the mudflat and *Roseovarius* sp. VA014 from the steel structure, were able to develop a stable single-species biofilm under dynamic conditions. Each biofilm had a specific structure. Interestingly, these strains were the four bacteria that displayed the thickest biofilms (more than 30 μm) on glass surfaces under static conditions (Fig. 3b).

## Discussion

In this work, we studied bacteria that inhabited two types of marine biofilms. Out of 156 isolates of our marine bacterial collection, only 15 strains from the mudflat biofilms and 10 strains from the corroded metallic structures were able to form single-species biofilms on polystyrene surfaces. This low number of biofilm forming bacteria could be explained by the experimental conditions. For instance, monospecies biofilms were performed, whereas in natural environments, the presence of different bacteria may help to build a biofilm. Moreover, the substratum we used differed from that of the original ecosystem, and the time we let for the bacteria to attach to their support was 2 h only. This step was particularly relevant for pioneer bacteria known to settle in few hours in marine biofilms [9]. Cell density, medium, temperature… may also influence the biofilm formation.

Among our marine cultured strains collection, the most important bacterial classes able to form a biofilm in microtiter polystyrene plates were Alphaproteobacteria (5 from the steel and 3 from the mudflat, of which 6 were Rhodobacterales), Flavobacteriia (4 from the mudflat and 2 from the steel, all being Flavobacteriales), Gammaproteobacteria (4 from the mudflat and 1 from the steel, of which 4 Alteromonadales) and Bacilli (3 from the mudflat and 1 from the steel, all being Bacillales). Only 1 strain from each kind of biofilm belonged to the Actinobacteria class. This is consistent with Dang et al. [19], who identified members of Alphaproteobacteria (mainly Rhodobacterales), Gammaproteobacteria (mainly Alteromonadales and Oceanospirillales), and Bacteroidetes (mainly Flavobacteriales) groups, as the most common and dominant surface colonizers in their study.

Out of the 8 Alphaproteobacteria able to build a biofilm on microtiter plates, we finally selected two strains belonging to the *Roseobacter* clade, *Roseobacter* sp. IV3009 and *Roseovarius* sp. VA014, for their capacity to also form a biofilm on glass surface under dynamic conditions. It is well known that the *Roseobacter* clade members are the dominant and ubiquitous primary surface colonizers whatever the type of surfaces, in temperate coastal waters (Pacific and Atlantic coasts) [5, 10, 11, 19]. Moreover, Dang et al. [19] suggested that *Roseobacter* were early steel surface colonizers, but also participated to the process of biofilm growth, while *Roseovarius* would only be pioneer surface colonizers. *Roseobacter* sp. IV3009 and *Roseovarius* sp. VA014 are therefore two interesting models of potential pioneering bacteria in marine biofilms.

Among the 5 Gammaproteobacteria able to form a biofilm on polystyrene, 4 were Alteromonadales, with 3 *Shewanella* and 1 *Alteromonas*. When Lee et al. [9] studied the succession of bacterial communities during the first 36 h of biofilm formation on acryl, glass and steel substratum in seawater, they observed that some species of Gammaproteobacteria, such as *Alteromonas*, were predominant during the first 9 h. *Shewanella* sp. was until now not described as a predominant bacterium in Atlantic marine biofilms, but was recently observed in early biofilms from Mediterranean Sea [14]. *Shewanella* sp*.* IV3014 was selected in this work for its ability to develop an original hairy biofilm with horizontal fibres under dynamic conditions.

We observed a very high diversity among the 6 Flavobacteriia strains able to adhere on polystyrene: they belong to 6 different genera. Previous works showed that bacteria of the Bacteroidetes phylum (containing the Flabovacteriia class) constituted a dominant and diverse bacterial group on carbon steel coupons, at all early immersion stages [16, 19], and it was suggested that different strains might be involved at different stages of the surface colonizing and development microbiota [19]. In our work, only one Flavobacteriia strain, *Flavobacterium* sp*.* II 2003, was finally able to form a biofilm in dynamic conditions. Both in static and dynamic conditions, *Flavobacterium* sp. II2003 displayed a very thick biofilm. It is known that pathogenic *Flavobacterium* species are responsible for great losses of fish in aquaculture farming worldwide [20, 21]. Aquaculture surfaces are easily colonized and persisting *Flavobacterium* sp. inhabiting biofilms might serve as a source of infection or reinfection [20]. Although *Flavobacterium* sp. are important pathogens in the aquaculture setting and have been detected in industrial, domestic, and medical environment biofilms, the manner in which they form biofilms has not been elucidated [20]. Therefore, the *Flavobacterium* sp*.* II2003 strain constitutes a very interesting model.

We detected 4 bacteria affiliated to Firmicutes (*Bacillus*) able to form biofilms on polystyrene. However, Firmicutes were identified as minor phyla found in biofilms formed on acryl, glass and steel substratum in seawater [9, 10, 22]. In our experiments, the Firmicutes then represent a high proportion compared to what occurs in natural environments. However, none of these strains was able to adhere to glass surfaces and they could not be retained as models. The same phenomenon was observed for the two Actinobacteria strains.

## Conclusions

In conclusion, this work allowed us to finally select four bacteria able to form a thick biofilm on polystyrene as well as glass surface under dynamic conditions: *Flavobacterium* sp. II2003, *Roseobacter* sp. IV3009, *Shewanella* sp*.* IV3014 from the mudflat biofilm, and *Roseovarius* sp. VA014 from the corrosion product-microorganism composite biofilm. Moreover, each of the four strains was able to develop a biofilm with a specific 3D structure. It will then be possible to accurately study potential pioneering bacteria in marine biofilms. Primary colonizers are known to be responsible for the initiation of biofouling and may cause various damages in maritime activities and industries. The paramount importance of the bacterial primary colonizers in surface community formation, dynamics, and function needs to be explored. In future studies, we will investigate the interactions between these high forming-biofilm bacterial models and other marine bacteria from the same ecosystems in order to better understand the initial stage of marine biofilm formation.

## Methods

### Bacterial strains isolation and culture media

A wide range of heterotrophic aerobic bacteria (156) was isolated from two marine biofilms. Benthic bacteria were collected from the intertidal temperate mudflat biofilm of Marennes-Oléron Bay (45°55’N, 01°06’W, Atlantic Coast of France), during three days at low tide in February and July 2008, at 2 h, 3 h and 4 h after emersion. Mudflat samples were collected using core diameter of 20 cm, and the top 2–3 mm was taken. After sampling, mudflat samples were carried to the laboratory at 4° C and immediately processed. The second source of bacteria was the biofilm associated with the corrosion products formed on carbon steel structures immersed in seawater [16]. Briefly, carbon steel coupons (70 × 70 × 6 mm) were immersed in a harbour of La Rochelle (Atlantic coast, France) for 1 week to 2 months at a constant depth of 1 m. The steel composition (98.2 % Fe, 0.122 % C, 0.206 % Si, 0.641 % Mn, 0.016 % P, 0.031 % S, 0.118 % Cr, 0.02 % Mo, 0.105 % Ni and 0.451 % Cu) was the same as that of the harbour metallic structures. At the end of the experiment, the corroded coupons were carried to the laboratory in sealed bags filled with seawater and immediately processed. The mudflat samples and biofilms scraped from the corroded coupons were resuspended in sterile artificial seawater (sea salts Sigma 35 g l^-1^) and inoculated on Marine Agar (Difco) supplemented with cycloheximide (Sigma 100 μg ml^-1^) to prevent eukaryotic growth. Bacterial isolates were obtained from the plates after incubation at 20° C in aerobic conditions. Strains were conserved as frozen stocks with 25 % glycerol at -80°C until further processing. For all subsequent tests, the strains were grown in Zobell broth (pastone Bio-Rad 4g l^-1^; yeast extract Bio-Rad 1g l^-1^; sea salts Sigma 30g l^-1^) at 22° C with shaking (150 rpm).

### DNA extraction and 16S rRNA gene sequencing

The isolated bacterial strains were identified by 16S rRNA gene sequencing. The genomic DNA of bacteria was extracted with the Genomic DNA from Tissue Kit (Macherey Nagel) from 1 to 5 ml of overnight culture. Amplification of about 1400 bp of the 16S rRNA gene was carried out using 50 ng of genomic DNA in a total volume of 50 μl. The reaction mixtures contained 0.2 μmol l^-1^ 16SUnivF (_5’_AGAGTTTGATCCTGGCTCA_3’_) and 16SUnivR (_5’_GGCTACCTTGTTACGACTT_3’_) primers, 3 mmol l^-1^ MgCl_2_, 320 μmol l^-1^of each dNTP, and 0.04 Taq polymerase Units (Fermentas), in the corresponding 1× buffer. Denaturation at 95° C for 2 min was followed by 30 cycles of amplification (92° C for 30 s, 54° C for 30 s, 72° C for 1 min 30). About 300 ng of each amplified DNA were sent to GenoScreen (Lille, France) for sequencing. The 16S rDNA sequences were compared with those in GenBank using the Blast software (National Institutes of Health, USA).

### Growth of biofilm on polystyrene surfaces (microtiter plates) under static conditions, crystal violet staining

The ability of the bacterial strains to form biofilms onto polystyrene was tested individually by cultivating each of them in 96-well microtiter plates (MICROTEST™ 96, Falcon) under static conditions and by crystal violet staining. The protocol used was a modified version of that described by O’Toole and Kolter [23]. Cells of an overnight bacterial culture were resuspended after 10 min of centrifugation at 7000 g in artificial seawater to a final optical density at 600 nm (OD_600_) of 0.25 and 150 μl of the resulting bacterial suspensions were loaded per well of the microtiter plates. After incubation for 2 h at 22° C, the wells were gently washed three times with artificial seawater. Thereafter, 150 μl of Zobell medium were transferred in each well and the plate was incubated at 22° C for 24 h. The planktonic fractions were transferred into a new microtiter plate and the absorbance was measured at 600 nm. The plates with biofilms were washed three times with artificial seawater. The biofilms were then stained with a 0.8 % crystal violet solution for 20 min. The wells were then rinsed with ultra-pure water until the wash-liquid was clear (10 times on average) and 150 μl of 96 % ethanol was added to solubilize the attached crystal violet from biofilms. Quantification was carried out by measuring the OD_595_. To be able to compare the results obtained with strains showing different growth speeds, the biofilm formation was expressed as the ratio of OD_595_ (cells grown in biofilm)/OD_600_ (planktonic cells). Assays were performed in triplicate for each strain.

### Growth of biofilm on glass surfaces under static conditions, fluorescence microscopy and image analyses

For each tested strain, cells of an overnight bacterial culture were resuspended after 10 min of centrifugation at 7000 g in artificial seawater to a final OD_600_ of 0.25. One ml of the resulting bacterial suspension was loaded in a compartment of Petri dishes (CellView diameter 35 mm, Greiner Bio-one) containing four compartments and a glass bottom. After incubation for 2 h at 22° C, the compartments were gently washed with artificial seawater and 1ml of Zobell medium was poured in each compartment. Biofilms were then grown for 24 h at 22° C. The surfaces were then rinsed with artificial seawater and biofilms were stained with 4 μg l^-1^ 4–6-diamidino-2-phenylindole (DAPI) in the dark for 20 min. After rinsing with ultra-pure water and drying, samples were analysed using a fluorescence microscope DMI6000B system (magnification 1000×, Leica Microsystems, Germany), over an average of 10 fields. The three dimensional (3D) structures were reconstituted by using IMARIS software. The percentages of colonized surface (%) were calculated using the NIH ImageJ software [24]. All experiments were performed in triplicate.

### Growth of biofilm on glass surface under dynamic conditions in three channel flow cell, confocal laser scanning microscopy and image analyses

Bacterial biofilms were grown on glass slides in three-channel flow cells (channel dimensions 1 by 4 by 40 mm, Technical University of Denmark Systems Biology, Denmark) [25]. The flow system was assembled, prepared and sterilized as described by Tolker-Nielsen and Sternberg [26]. The substratum consisted in a microscope glass coverslip (24 × 50 st1, KnittelGlasser, Germany). Flow cells were inoculated with overnight bacterial cultures diluted in artificial seawater to a final OD_600_ of 0.1. Bacteria were allowed to attach during 2 h at 22° C without medium flow. The channels were then washed by applying a flow of artificial seawater for 15 min at a rate of 2 ml h^-1^ to remove planktonic cells. Biofilm growth was then performed under a constant flow of Zobell (2 ml h^-1^) for 24 h at 22° C. Microscopic observations were performed by confocal laser scanning microscopy using a TCS-SP2 system (Leica Microsystems, Germany). The 2-h attached cells and the biofilms formed after 24 h on the glass surface were observed by staining bacteria with 5 μmol l^-1^ Syto 61 red. Images were obtained using the Leica confocal software. The surface coverages after the 2 h adhesion step were evaluated using the ImageJ software. The biofilm stacks were analysed with the COMSTAT software (developed in MATLAB, [27]) to estimate the maximal and average thicknesses (μm) and the biovolume (μm^3^ μm^-2^). The values were calculated from three independent experiments from which a total of 15 image stacks were obtained.

### Statistical analyses

The standard deviations were calculated using Matlab software (Mathworks Inc., Natick, USA). The statistical analyses were determined by the Student *t*-test and considered as significant if *p* values are < 0.05.
